# Phenotypic manifestation of Brugada syndrome after living donor liver transplantation accompanied by normalization of sex hormone levels

**DOI:** 10.1016/j.hrcr.2026.06.004

**Published:** 2026-06-11

**Authors:** Eiichiro Nakagawa, Hideyuki Komekado, Mami Hata, Nobuhiro Sato, Tosinori Makita

**Affiliations:** 1 Department of Cardiology, Osaka Saiseikai Izuo Hospital, Osaka City, Osaka, Japan; 2 Department of Gastroenterology and Hepatology, Japanese Red Cross Osaka Hospital, Osaka City, Osaka, Japan; 3 Department of Clinical Laboratory, Japanese Red Cross Osaka Hospital, Osaka City, Osaka, Japan; 4 Department of Cardiovascular Internal Medicine, Yao Tokushukai General Hospital, Yao City, Osaka, Japan

**Keywords:** Brugada syndrome, Hepatic dysfunction, Liver transplantation, Sex hormones, Testosterone, Estrone

**Key Teaching Points**

• To date, no reports have described an association between liver dysfunction and Brugada syndrome. This report presents the first documented case in which the manifestation of Brugada syndrome emerged only after liver transplantation following 18 years of liver disease.
• In addition to improvements in hepatic function, nutritional status, feminization, and sex hormone concentrations normalized after liver transplantation. A diagnostic Brugada electrocardiographic pattern was also consistently observed for more than 3 years thereafter.
• Sex hormones have been shown to influence electrocardiographic morphology in patients with Brugada syndrome, and sex-related differences in phenotypic expression are well recognized despite its autosomal dominant inheritance. Sex hormones might play an important role in the phenotypic manifestation of Brugada syndrome after liver transplantation.

## Introduction

Brugada syndrome was reported for the first time in 1992^1^ and is associated with characteristic ST-segment elevation in the right precordial leads (V_1_–V_3_) in the absence of any demonstrable structural heart disease as well as sudden cardiac death due to ventricular fibrillation. A diagnosis of Brugada syndrome is established when spontaneous coved-type ST-segment elevation (>0.2 mV at the J point) is observed in 1 or more right precordial leads (V_1_–V_3_).^2^

Brugada syndrome has been reported to occur 8–10 times more frequently in men,^3^ and it is currently considered to be phenotypically expressed through an oligogenic or polygenic etiology.^4^ Di Diego et al^5^ suggested that the predominance of the Brugada phenotype in male patients results from the presence of a more prominent transient outward current in male right ventricular (RV) epicardial myocytes compared with female myocytes, which contributes to the deeper notch in phase 1 of the action potential of male myocytes. In contrast, other evidence raises the possibility that the male hormone, testosterone, plays an important role in the phenotypic manifestations of Brugada syndrome, resulting in the male predominance.^6^^,^^7^ In addition, it is suggested that female sex hormones might affect the phenotypic manifestation of Brugada syndrome in several studies.^8^, ^9^, ^10^ However, the precise mechanism underlying the sex differences in the manifestations of Brugada syndrome remains unclear. In this report, we present an interesting case in which spontaneous coved-type ST-segment elevation emerged for the first time, along with the increased serum testosterone and decreased serum estrone levels after liver transp lantation.

## Case report

**Figure 1:**
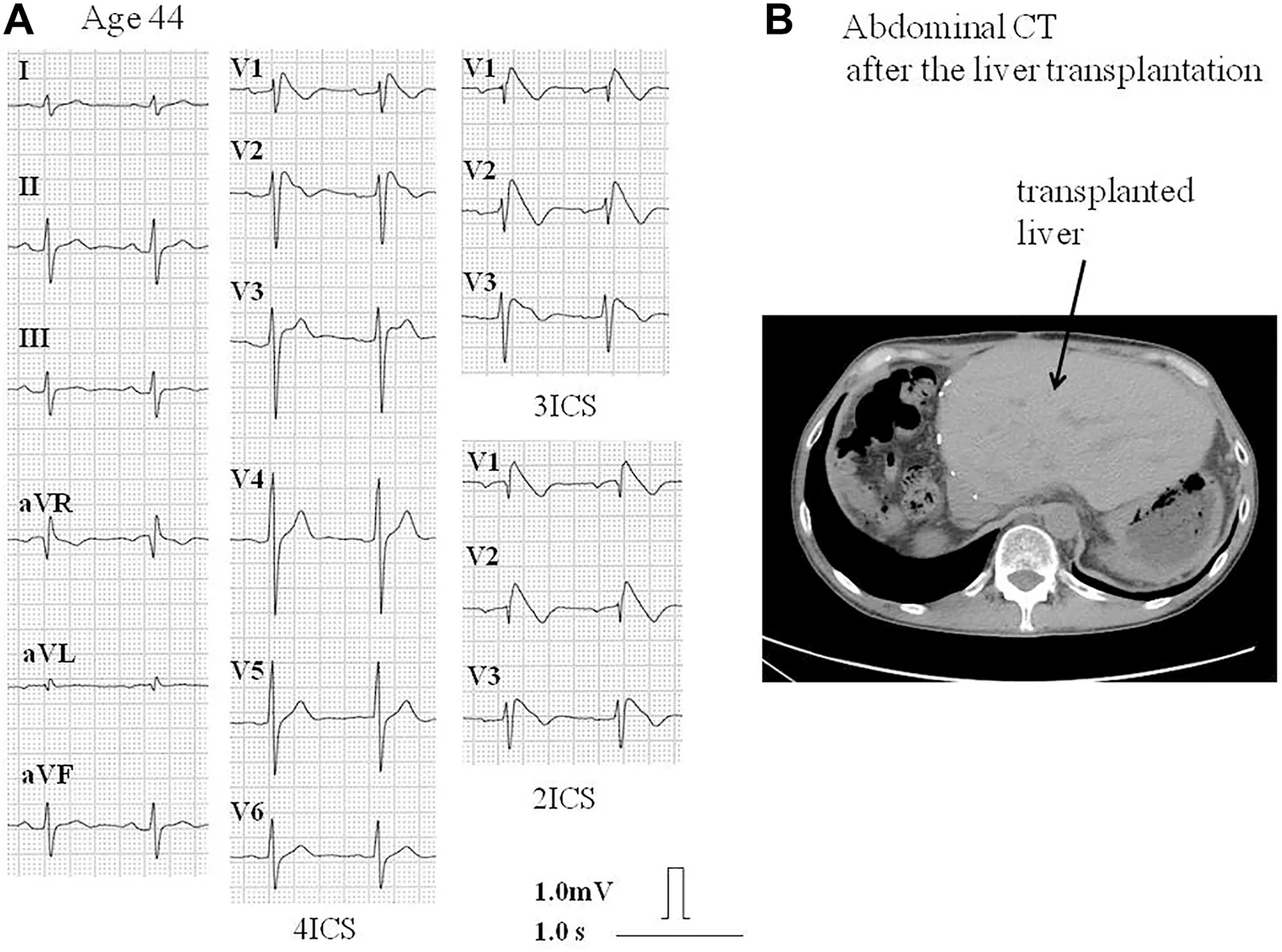
**A:** 12-lead electrocardiogram recorded 17 months after liver transplantation at the age of 44. Coved-type ST-segment elevation was observed in leads V_1_ and V_2_, with a J-point amplitude of 0.35 and 0.38 mV, respectively, at the fourth intercostal space (4ICS), the usual recording site. Coved-type ST-segment elevation was also observed in leads V_1_, V_2_, and V_3_ at the second and third intercostal spaces (2ICS and 3ICS). **B:** Abdominal computed tomography (CT) performed after liver transplantation.

A 44-year-old man was referred to our outpatient clinic for cardiac evaluation, in whom coved-type ST-segment elevation in the right precordial leads with a J-point amplitude of 0.35 mV in lead V_1_ and 0.38 mV in lead V_2_ was documented for the first time in his life 17 months after undergoing living donor liver transplantation (Figure 1). He had not experienced episodes of syncope, agonal respiration during nighttime sleep, or ventricular fibrillation. He had no family history of Brugada syndrome or sudden cardiac death. An initial physical examination revealed normal findings, except for abdominal surgical scars. Further examinations, including a chest radiograph and echocardiogram, revealed no evidence of structural heart disease or any other abnormalities. Finally, the diagnosis of Brugada syndrome was established according to the J-wave syndromes expert consensus conference report.^2^

**Figure 2:**
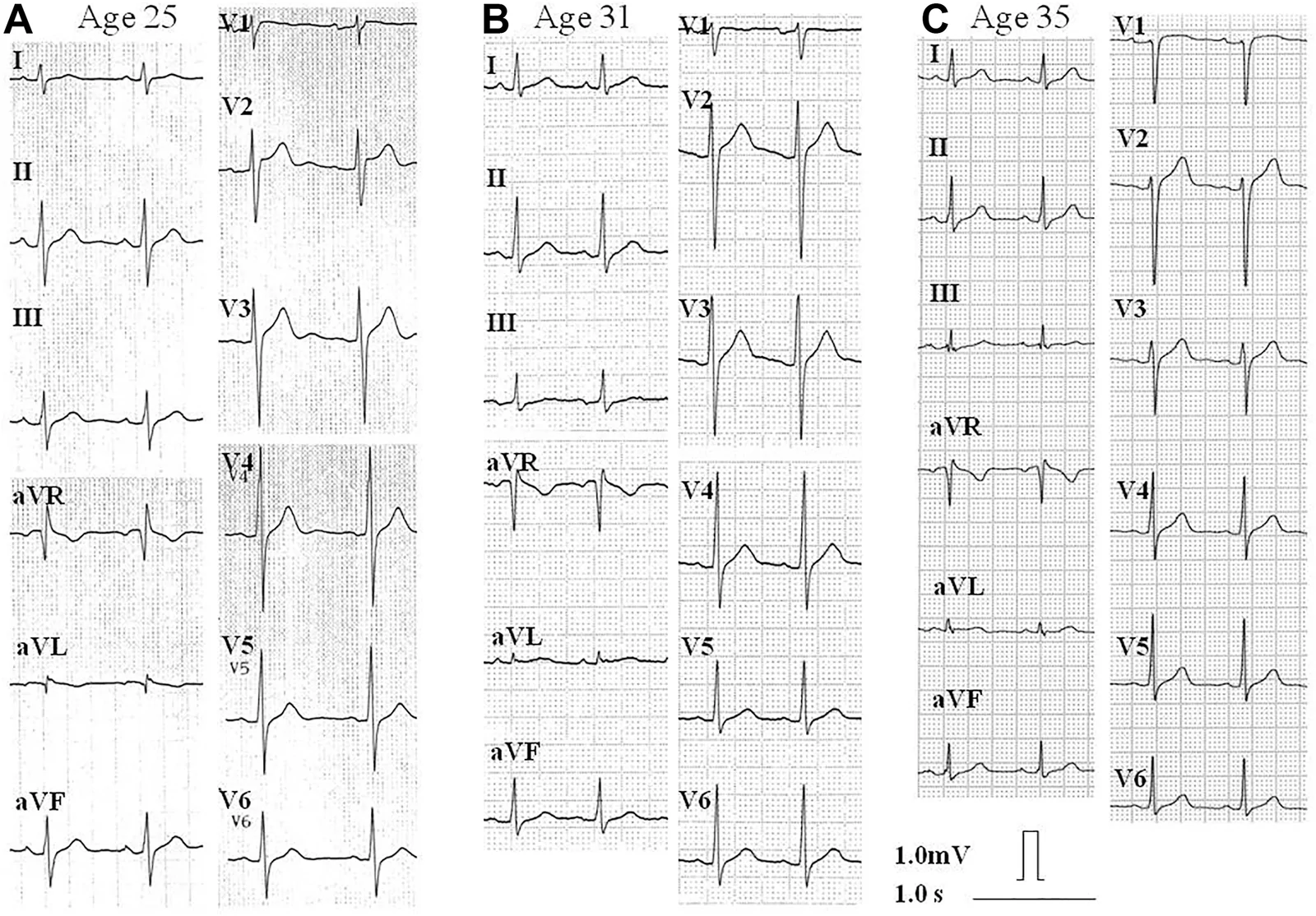
**A:** 12-lead electrocardiogram (ECG) recorded at the age of 25, when liver dysfunction first manifested. No significant morphological changes were observed in the right precordial leads, except for J-point elevation in leads V_1_, V_2_, and V_3_, with a J-point amplitude of 0.12, 0.21, and 0.20 mV, respectively. **B:** 12-lead ECG at the age of 31 during an episode of esophageal variceal rupture. Compared to the ECG shown in panel A, J-point amplitudes in leads V_1_, V_2_, and V_3_ were decreased, measuring 0.06, 0.12, and 0.09 mV, respectively. **C:** 12-lead ECG at the age of 35, when the patient was diagnosed with liver cirrhosis. No significant morphological changes were observed in the right precordial leads; J-point amplitudes in leads V_1_, V_2_, and V_3_ were 0.09, 0.12, and 0.10 mV, respectively.

**Figure 3:**
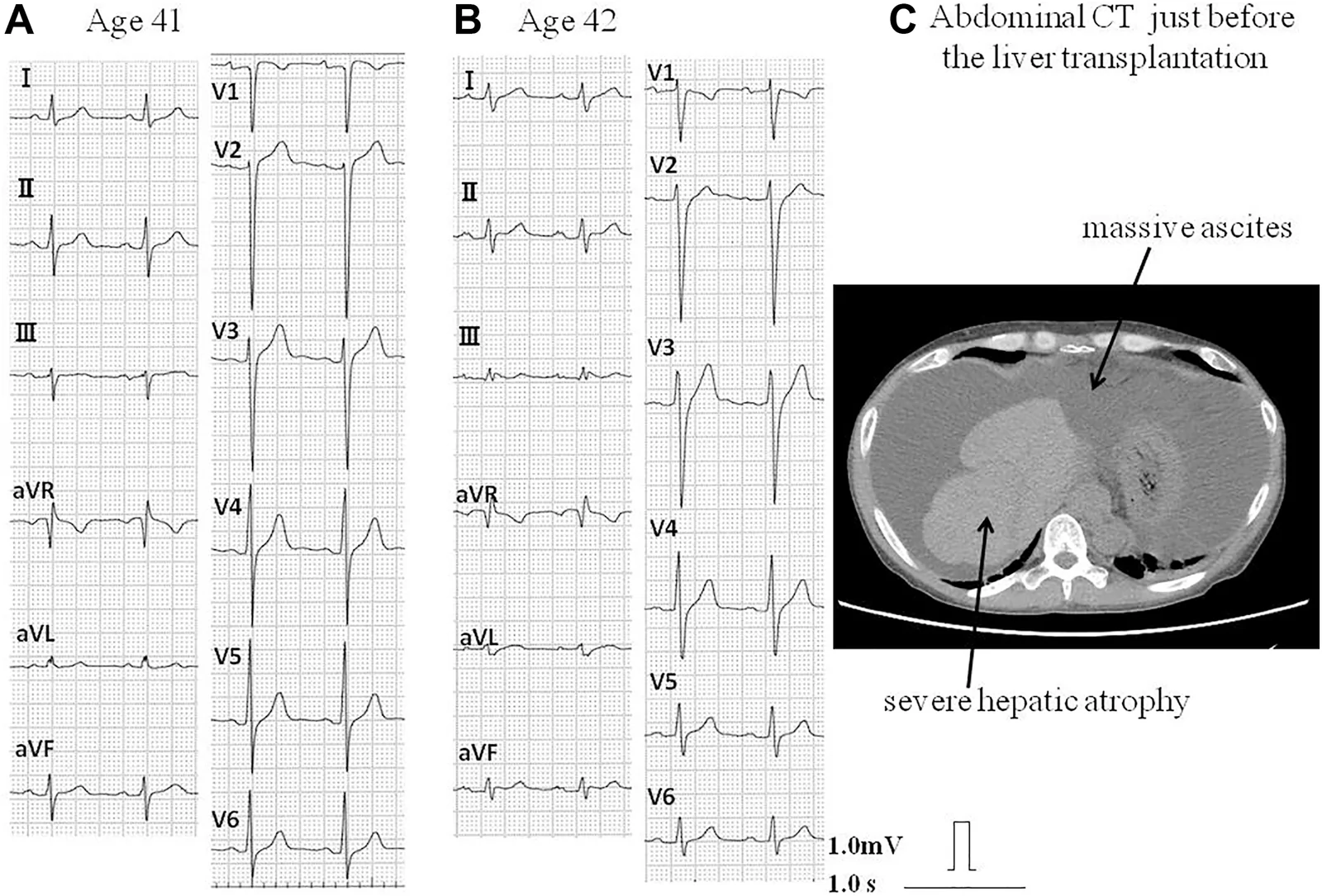
**A:** 12-lead electrocardiogram (ECG) recorded at the age of 41, when type 3 Brugada ECG with coved-type ST-segment elevation was rarely observed. **B:** 12-lead ECG at age 42 recorded 2 months before liver transplantation, showing slight QRS prolongation with a QRS duration of 120 ms. **C:** Abdominal computed tomography (CT) performed just before liver transplantation, showing massive ascites and severe hepatic atrophy.

**Figure 4:**
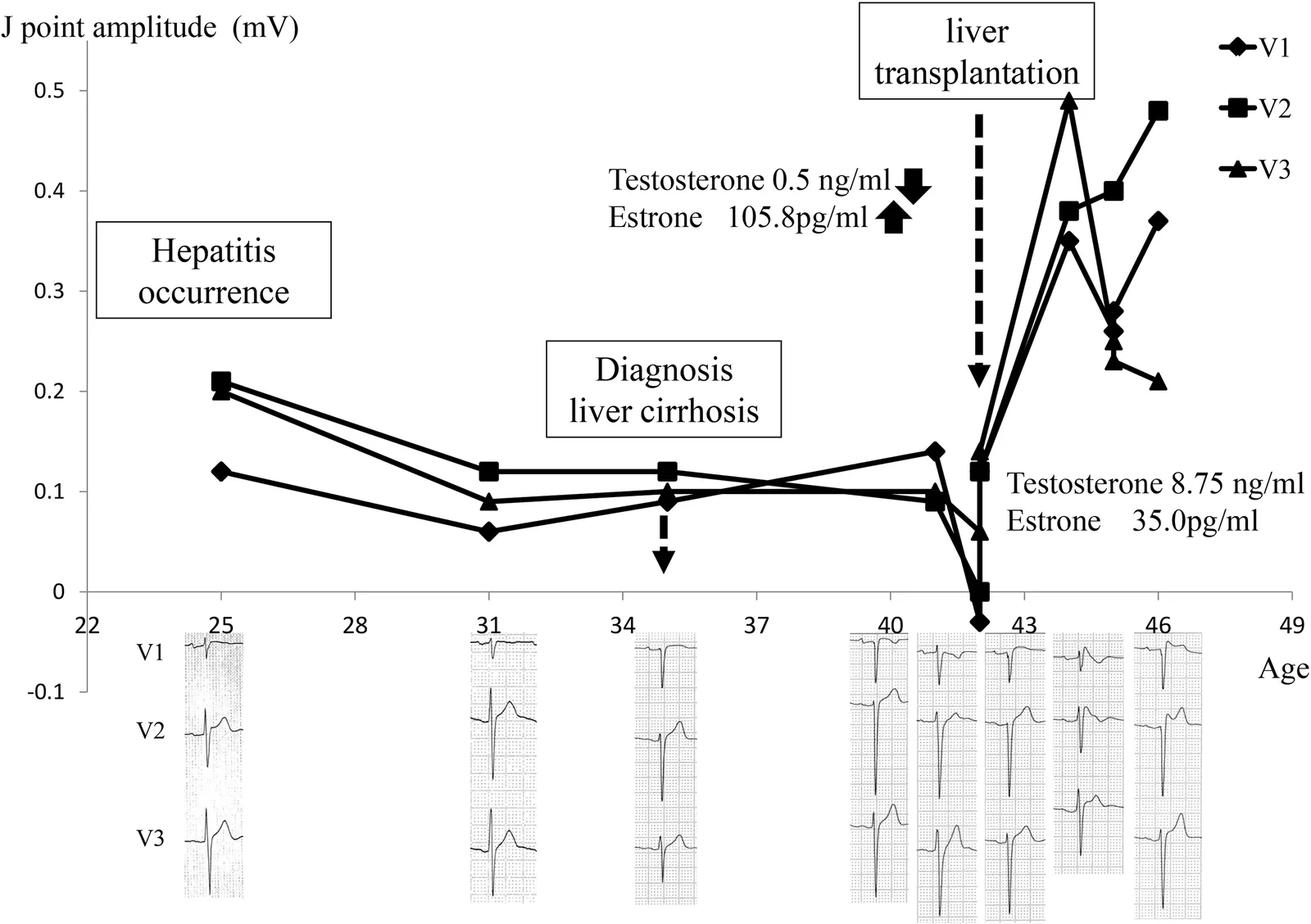
Temporal trends in J-point amplitude and morphological changes in the electrocardiogram in leads V_1_, V_2_, and V_3_ at the fourth intercostal space over the years. A trend of slight downward displacement of the J-point amplitude in leads V_2_ and V_3_ was observed before liver transplantation. J-point amplitude elevation has been consistently observed in leads V_1_, V_2_, and V_3_ after liver transplantation. Diagnostic coved-type ST-segment elevation was observed 17 months after liver transplantation, together with normalization of serum testosterone and estrone concentrations.

**Figure 5:**
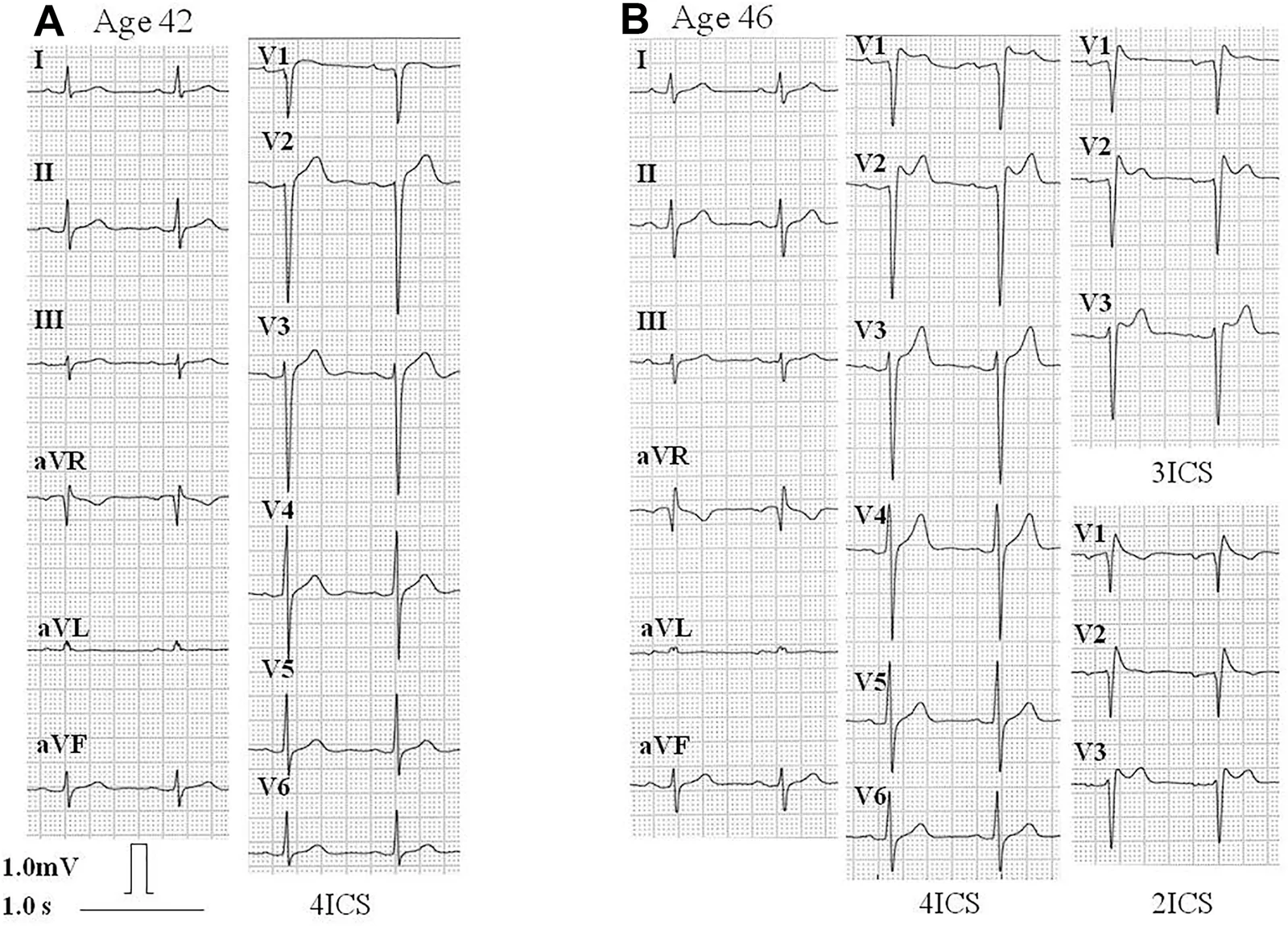
**A:** 12-lead electrocardiogram (ECG) recorded at the age of 42, 2 months after liver transplantation. Although J-point amplitude elevation was observed in leads V_1_, V_2_, and V_3_ compared to the pretransplant period, no diagnostic coved-type ST-segment elevation was noted. **B:** 12-lead ECG at the age of 46, 31 months after liver transplantation. Although only saddleback-type ST-segment elevation was observed in the right precordial leads at the fourth intercostal space (4ICS), coved-type ST-segment elevation with J-point amplitude exceeding 0.20 mV was observed in lead V_1_ at the third intercostal space (3ICS) and in leads V_1_ and V_2_ at the second intercostal space (2ICS).

He had been suffering from hepatic disease of unknown etiology since he was 25 years old when his liver had already exhibited advanced morphological changes, often described as “potato-like liver,” and the 12-lead electrocardiogram (ECG) showed no morphological abnormalities except for slight J-point amplitude elevation in leads V_1_, V_2_, and V_3_ (Figure 2). Since then, he had been treated for his hepatic disease by 2 university hospitals and then our hospital sequentially, but his hepatic function gradually declined. The patient experienced an episode of rupture of esophageal varices at the age of 31, and he was subsequently diagnosed with liver cirrhosis at the age of 35, with a Child-Pugh score of 7. There had been no significant morphological changes on 12-lead ECGs, except for the rare appearance of type 3 coved-type ST-segment elevation in lead V_1_ (Figures 2 and 3), but a trend of slight downward displacement of the J-point amplitude in leads V_2_ and V_3_ was observed (Figure 4). At the age of 43, his liver function had markedly deteriorated with a Child-Pugh score of 14 and he presented with the end-stage symptoms of hepatic failure, such as massive ascites, gynecomastia, jaundice, and skeletal muscle depletion, as well as a very reduced serum testosterone concentration of 0.5 ng/mL, an increased estrone concentration of 105.8 pg/mL, and a normal range of estradiol concentration of 20.3 pg/mL (Figure 3). He then underwent living donor liver transplantation (Figure 1). 2 months after liver transplantation, no significant ECG changes were found (Figure 5). However, 17 months later, typical coved-type ST-segment elevation with a J-point amplitude exceeding 0.35 mV appeared in leads V_1_ and V_2_ at the fourth intercostal space and in leads V_1_, V_2_, and V_3_ at the third and second intercostal spaces spontaneously (Figure 1). Subsequently, the diagnosis of Brugada syndrome was established. Laboratory testing revealed that the serum testosterone concentration increased to slightly above the normal range (8.75 ng/mL) and the serum estrone concentration decreased to the normal range (35.0 pg/mL), together with the disappearance of hepatic failure symptoms, such as massive ascites and gynecomastia. Chest radiography and computed tomography revealed that the anatomical relationship of the heart to the anterior thoracic wall remained unchanged before and after liver transplantation. Treadmill exercise testing and a 12-lead 24-hour Holter recording were performed after the manifestation of Brugada syndrome. Diagnostic spontaneous coved-type ST-segment elevation in the right precordial leads had been continuously observed at a heart rate of approximately 100–110 beats/min during exercise and daily activities.

Although diagnostic spontaneous coved-type ST-segment elevation in the right precordial leads had never been documented from the age of 25 until the recovery of hepatic function, it has been consistently observed in leads V_1_ and V_2_ at the usual fourth intercostal space or higher intercostal spaces during the 3-year follow-up period after the manifestation of Brugada syndrome (Figure 5). A persistent upward displacement of J-point amplitude exceeding 0.20 mV has also been concurrently observed in leads V_1_, V_2_, and V_3_ at the usual fourth intercostal space (Figure 4). No medication reported to affect the ECG configuration in patients with Brugada syndrome was administered at any time during the patient’s clinical course.

A Brugada syndrome genetic panel including preliminary evidence genes such as *CACNA1C*, *CACNA2D1*, *CACNB2*, *HCN4*, *KCND3*, *KCNE3*, *KCNE5*, *KCNH2*, *KCNJ8*, *PKP2*, *SCN10A*, *SCN1B*, *SCN2B*, *SCN3B*, *SCN5A*, and *TRPM4* was tested. No pathogenic variants were detected in the patient.

## Discussion

The major finding in this case is that diagnostic coved-type ST-segment elevation in the right precordial leads was manifested for the first time after living donor liver transplantation in a male patient with Brugada syndrome, which has been consistently observed thereafter, together with the improvement of hepatic function and the normalization of serum concentrations of testosterone and estrone. To our knowledge, this is the first case indicating a possible relationship between hepatic dysfunction and the manifestation of Brugada syndrome.

Although it has been reported that QT prolongation is observed in patients with liver cirrhosis,^11^ there have been no reports describing the effect of hepatic dysfunction on the morphological changes of ECG in patients with Brugada syndrome. In contrast, it is well known that hepatic impairment leads to alterations in circulating sex hormone levels—characterized by elevated estrone and decreased testosterone serum concentrations—which are gradually restored to physiological levels after liver transplantation over a period of several months.^12^, ^13^, ^14^

In our case, the serum testosterone level immediately before liver transplantation was very low and comparable to the levels typically observed after surgical castration and the serum estrone level was approximately 3 times higher than the upper limit of the normal range for healthy middle-aged men. Normalization of serum testosterone and estrone levels was observed 17 months after liver transplantation. Several clinical studies have suggested that sex hormones might contribute to the phenotypic manifestation and sex differences in patients with Brugada syndrome.^6^^,^^15^^,^^16^ Matsuo et al^6^ reported 2 male cases, in whom surgical castration led to normalization of coved-type ST-segment elevation in the right precordial leads, which had been consistently observed for more than 18 years before the operation. The regression of ECG features occurred in both cases within a period of 2 years after the operation. There are 2 prior case reports of female patients reporting unmasking of Brugada syndrome during female-to-male transition with testosterone administration.^15^^,^^16^ Especially in 1 patient, diagnostic coved-type ST-segment elevation in the right precordial leads was no longer observed after temporary suspension of testosterone. When testosterone was reintroduced, it appeared on the ECG again.^15^

Yan and Antzelevitch^17^ proposed that the depression or loss of the action potential dome in the RV epicardium, resulting in a transmural voltage gradient, may underlie ST-segment elevation in the right precordial leads in Brugada syndrome. The maintenance of the action potential dome is dependent on the balance of ionic currents active during phase 1 of the action potential (principally transient outward current, sodium channel current, and calcium channel current).^17^ In addition, RV conduction slowing, which is attributable to a reduction in inward depolarization currents and potentially exacerbated by structural abnormalities in the pericardium of the RV outflow tract, has been implicated in the ECG features of Brugada syndrome.^18^ Several experimental studies have demonstrated that testosterone enhances outward potassium currents, including the rapidly activating component and the slowly activating component of the delayed rectifier potassium current and the inward rectifier potassium current,^19^, ^20^, ^21^ and decreases the inward L-type calcium current.^21^ Moreover, high doses of testosterone have been shown to induce myocardial hypertrophy and fibrosis.^22^ Shimizu et al^7^ reported that male patients with Brugada syndrome had significantly higher testosterone levels than those in control male subjects. These findings suggest that testosterone may contribute to the manifestation of ST-segment elevation in the right precordial leads and the phenotypic expression of Brugada syndrome, potentially explaining the male predominance of Brugada syndrome.

On the contrary, there are several studies that indicate that female hormones are attributable to the male predominance and phenotypic manifestation of Brugada syndrome. In a cohort study that analyzed 15 young female and 42 male probands with Brugada syndrome, who were younger than 20 years, the number of new diagnoses and the incidence of lethal arrhythmic events decreased to 0% only in female patients with Brugada syndrome after puberty. In addition, 56% of female patients showed attenuation of the diagnostic coved-type ST-segment elevation after puberty, which were not found in male patients.^8^ Other studies have shown that the initial onset of clinical events in female patients with Brugada syndrome occurs at a later age compared with male patients, with a peak incidence around 48 years, which temporally coincides with the average age of menopausal onset in women.^9^^,^^10^ Although there have been no studies examining the effect of estrone on the action potential of myocytes and surface ECG, an experimental study showed that estradiol, another female hormone, decreases the rapidly activating component and the slowly activating component of the delayed rectifier potassium current and the inward L-type calcium current in ventricular myocytes, which might affect the action potential in the RV epicardium.^23^ Considering these studies mentioned above and the measured male and female sex hormone levels in our patient, the lack of clinical manifestations before liver transplantation may be attributable to a low serum testosterone concentration, elevated estrone levels, or a synergistic effect of both. Conversely, the manifestation of Brugada syndrome after liver transplantation might be induced by elevated serum testosterone levels, reduced serum estrone levels, or a combination of both.

Although diagnostic coved-type ST-segment elevation had never been observed over an 18-year period from the onset of liver dysfunction at the age of 25 until living donor liver transplantation at the age of 43 in our patient, gradual attenuation of the J-point amplitude in the right precordial leads was observed in parallel with the progression of hepatic dysfunction before liver transplantation (Figure 4). The change in J-point amplitude might be caused by a downward trend in testosterone levels or an upward trend in estrone levels, which had been reported to progress along with the worsening of liver function.^24^ Conversely, a persistent upward displacement of J-point amplitude in the right precordial leads after liver transplantation might be attributable to elevated serum testosterone levels, reduced serum estrone levels, or a combination of both (Figure 4).

Autonomic dysfunction in patients with cirrhosis has been described, characterized by reduced vagal tone and impaired sympathetic conduction to the heart.^25^ The morphology of ST-segment elevation in patients with Brugada syndrome is known to fluctuate spontaneously, and in addition to sex hormones, various factors including autonomic activity, exercise and food intake have been reported to influence both the degree and the configuration of ST-segment elevation.^26^, ^27^, ^28^ ST-segment elevation in patients with Brugada syndrome was augmented by selective stimulation of α-adrenoceptors or muscarinic receptors, but was mitigated by β-adrenoceptor stimulation.^29^ In the present case, exercise stress testing and a 12-lead 24-hour Holter recording were performed after the manifestation of Brugada syndrome. During exercise, coved-type ST-segment elevation diminished but returned to the same morphology within several minutes during the recovery phase. Notably, coved-type ST-segment elevation with a J-point amplitude of 0.25 mV persisted even at a heart rate of 115 beats/min, a condition in which sympathetic activity is increased and parasympathetic activity is decreased. Diagnostic coved-type ST-segment elevation in the right precordial leads was also continuously observed at a heart rate of approximately 100–110 beats/min in daily activities. In contrast, during the 18-year course of liver disease before transplantation, diagnostic coved-type ST-segment elevation had never been observed on ECGs recorded at a heart rate of approximately 70 beats/min. Even in the presence of hepatic dysfunction, sympathetic activity at a heart rate of 70 beats/min was presumed to be lower—and parasympathetic nerve activity higher—than that during exercise. Although autonomic activity fluctuates dynamically in daily life and was indeed observed to influence ECG morphology after the manifestation of Brugada syndrome in this case, it is plausible that sex hormones play a main role in its phenotypic expression. This is because circulating testosterone and estrone levels remain relatively stable throughout the day compared with autonomic activity, and the temporal pattern of their changes appears to correlate more closely with the ECG alterations observed over a follow‑up period of more than 20 years (Figure 4). However, because no studies have examined the effects of sustained alterations in autonomic activity on ECG morphology in patients with Brugada syndrome, it remains impossible to rule out the possibility that such autonomic fluctuations contributed to the ECG findings observed in this case. Further investigations are required to elucidate the mechanisms underlying the manifestation of Brugada syndrome after liver transplantation.

## Conclusion

In this case report, we describe the phenotypic manifestation of Brugada syndrome, which was strongly suggested to have been provoked by liver transplantation, accompanied by the normalization of male and female sex hormone levels in a male patient with liver cirrhosis.

## Disclosures

The authors have no conflicts of interest to disclose.
